# Spotlight on the impact of viral infections on Hematopoietic Stem Cells (HSCs) with a focus on COVID-19 effects

**DOI:** 10.1186/s12964-023-01122-3

**Published:** 2023-05-08

**Authors:** Kamyar Nasiri, Saman Mohammadzadehsaliani, Hadis Kheradjoo, Alireza Mohammadzadeh Shabestari, Parisa Eshaghizadeh, Azin Pakmehr, Marwa Fadhil Alsaffar, Bashar Zuhair Talib Al-Naqeeb, Saman Yasamineh, Omid Gholizadeh

**Affiliations:** 1 Department of Dentistry, Islamic Azad University, Tehran, Iran; 2 Ophthalmology Department, Buraimi Hospital, Buraimi, Oman; 3 Laboratory Department, Buraimi Hospital, Buraimi, Oman; 4 Department of Dental Surgery, Mashhad University of Medical Sciences, Mashhad, Iran; 5 Department of Dental Surgery, Tabriz University of Medical Sciences, Tabriz, Iran; 6 Medical Doctor, Tehran University of Medical Science, Tehran, Iran; 7 Medical Laboratories Techniques Department / AL-Mustaqbal University College, 51001 Hillah, Babil, Iraq; 8 Anesthesia Technology Department, Al-Turath University College, Al Mansour, Baghdad, Iraq; 9 Research Center for Clinical Virology, Tehran University of Medical Sciences, Tehran, Iran; 10 Department of Bacteriology and Virology, Faculty of Medicine, Tabriz University of Medical Sciences, Tabriz, Iran

**Keywords:** Hematopoietic stem cells, Viral infection, SARS-CoV-2, Hematopoietic system, HSC transplantation

## Abstract

Hematopoietic stem cells (HSCs) are known for their significant capability to reconstitute and preserve a functional hematopoietic system in long-term periods after transplantation into conditioned hosts. HSCs are thus crucial cellular targets for the continual repair of inherited hematologic, metabolic, and immunologic disorders. In addition, HSCs can undergo various fates, such as apoptosis, quiescence, migration, differentiation, and self-renewal. Viruses continuously pose a remarkable health risk and request an appropriate, balanced reaction from our immune system, which as well as affects the bone marrow (BM). Therefore, disruption of the hematopoietic system due to viral infection is essential. In addition, patients for whom the risk-to-benefit ratio of HSC transplantation (HSCT) is acceptable have seen an increase in the use of HSCT in recent years. Hematopoietic suppression, BM failure, and HSC exhaustion are all linked to chronic viral infections. Virus infections continue to be a leading cause of morbidity and mortality in HSCT recipients, despite recent advancements in the field. Furthermore, whereas COVID-19 manifests initially as an infection of the respiratory tract, it is now understood to be a systemic illness that significantly impacts the hematological system. Patients with advanced COVID-19 often have thrombocytopenia and blood hypercoagulability. In the era of COVID-19, Hematological manifestations of COVID-19 (i.e., thrombocytopenia and lymphopenia), the immune response, and HSCT may all be affected by the SARS-CoV-2 virus in various ways. Therefore, it is important to determine whether exposure to viral infections may affect HSCs used for HSCT, as this, in turn, may affect engraftment efficiency. In this article, we reviewed the features of HSCs, and the effects of viral infections on HSCs and HSCT, such as SARS-CoV-2, HIV, cytomegalovirus, Epstein-Barr virus, HIV, etc.

Video Abstract

**Supplementary Information:**

The online version contains supplementary material available at 10.1186/s12964-023-01122-3.

## Introduction

The hematopoietic system is in charge of producing new blood cells and their fundamental tasks, such as carrying oxygen (in the form of erythrocytes), stopping bleeding (in the form of platelets), and protecting the body from infection (in the form of white blood cells, including leukocytes (myeloid and lymphoid cells)). Recent estimates suggest that under homeostatic conditions (steady-state hematopoiesis), adult BM produces 2–6 × 10^10^ cells/kg/d in mice and 5–7 × 10^10^ cells/kg/d in humans. However, this number can increase several folds upon stress demands, such as infection (demand-adapted or "emergency" hematopoiesis) [1]. Pluripotent hematopoietic progenitors keep blood cells in circulation. These hematopoietic stem cells (HSCs) are uncommon pluripotent cells in the BM that express CD34 surface molecules and have the potential to develop into erythrocytes, granulocytes, monocytes, megakaryocytes, and lymphocytes [2]. After an infection or inflammation has caused damage to the hematopoietic system, HSCs play a crucial role in facilitating the repair and restoration of the system. It is uncertain whether the functional impairment of HSCs caused by these stresses persists beyond the period of inflammatory exposure [3]. To sustain their self-renewal ability and long-term maintenance, which is crucial for hematological system homeostasis, HSCs predominantly reside in a cell-cycle quiescence state when they are not actively being used. Abnormal hematopoiesis (such as myelodysplastic syndrome and bone marrow failure syndromes) and the development of leukemia may occur from a dysfunctional HSC. Cell-cycle regulators, transcription factors, epigenetic factors, and niche factors are only some of the examples of internal molecular networks and extrinsic signals that govern HSC quiescence [4]. New classes of hematopoietic stem and progenitor cells (HSPCs) with more limited self-renewal, proliferation, and differentiation potential are generated when these cells either die or commit to differentiation programs. A delicate balancing act between self-renewal and differentiation is required for lifelong, stable, and multilineage hematopoiesis, which is directed by both internal and external regulatory mechanisms [5].

After an allogeneic stem cell transplantation (SCT), viral infections are a significant source of morbidity and death. The risk of developing a severe viral infection increases following haploidentical SCT compared to other types of SCT, including those performed on related or unrelated donors. The central defensive mechanisms against exogenous viral infection are B-cell function and particular antibodies, which lowers the likelihood of reinfection in those already seropositive. However, T-cell activity, namely cytotoxic T-cell function, is the primary mechanism for avoiding severe viral illness as well as for controlling viruses like herpesviruses that might induce latency and then reactivate in an immunocompromised person. Furthermore, since specific antibody loss happens often following allogeneic SCT, it increases the risk of reinfection with previously encountered viruses such as measles and varicella-zoster virus (VZV). It allows the reactivation of antibodies-controlled viruses such as hepatitis B virus (HBV) [6]. Chronic viral infections typically appear clinically at the level of blood cell production. We investigated viruses that can cause BM illnesses such as aplastic anemia, pancytopenia, hemophagocytic lymph histiocytosis, lymphoproliferative disorders, and cancer. In addition, HSPCs and the surrounding tissue are vulnerable to the direct and indirect harm that viral infections may produce. Certain viruses, such as parvovirus B19, may infect HSPCs directly, causing changes in BM production, albeit this is rare and depends on the virus's tropism and life cycle. However, the intricate connections among viruses, HSPCs, and the BM microenvironment are poorly understood now. CMV is an example of a virus that may cause prolonged latent infection with no overt BM disease since it can infect both stroma and HSPC. Temporary aplasia is often seen in the early stages of a viral infection, and it is thought that this is due in part to the action of IFN-I and the fact that direct viral infection leads to the depletion of both HSPCs and stromal cells [7].

COVID-19, caused by the severe acute respiratory syndrome coronavirus 2 (SARS-CoV-2), became a worldwide pandemic. SARS-CoV-2 is a positive-sense, RNA virus that is enveloped [8, 9]. In acute cases, SARS-CoV-2 may lead to acute respiratory distress syndrome (ARDS) and acute lung injury (ALI). Flu-like symptoms, such as a high temperature, persistent coughing, weakness, chest discomfort, shortness of breath, delirium, a hoarse voice, a lack of appetite, diarrhea, and a diminished sense of taste and smell, are all possible side effects of COVID-19. However, most infected individuals show no signs of illness [9, 10]. There is also growing evidence that severe COVID-19 disease causes damage to hematopoietic stem/progenitor cells (HSPCs) and endothelial progenitor cells (EPCs). Anemia, lymphopenia, and thrombocytopenia have all been described in patients infected with SARS-CoV-2 [11]. Additionally, SARS-CoV-2 directly infects erythroid progenitor cells, disrupts the homeostasis of hemoglobin, and exacerbates COVID-19 illness. Red blood cell precursors are thought to be a direct target of SARS-CoV-2, and it has been hypothesized that this virus's induction of dysregulation in hemoglobin and iron metabolism is a factor in the severe systemic course of COVID-19 [12].

HSCs are great candidates for gene therapy because of the relative easiness with which they can be manipulated and their capability to repopulate the whole hematopoietic system for the life of a patient [13, 14]. New advances in gene therapy have resulted in research toward its use for ex vivo gene editing in HSCs, which may have benefits compared to combining viral-vector-interceded gene addition. This landscape will offer the early technique currently being utilized for gene editing of HSCs for clinical usages and gene addition using combining viral vectors, and also discuss the present situation of gene correction in human HSCs for autologous transplantation [15]. HSCs gene therapy is a perfect treatment method for viral infections. For example, various primary phase clinical evaluations have shown the harmlessness and possibility of stem cell gene therapy for HIV-1/AIDS. In addition, HSC gene therapy utilizing a released SARS-CoV-2 decoy receptor protein (sACE2-Ig) would involve a one-time intervention leading to long-time preservation versus airway infection, viremia, and extrapulmonary symptoms [16–18].

Therefore, this review first provides a brief overview of the various characteristics of HSCs, and how HSCs respond to viral infections to set the stage for a discussion of how SARS-CoV-2 infection affects HSCs and HSCT.

## Hematopoietic stem cells (HSCs)

Anemia, immunodeficiency, clonal hematopoiesis, and hematological malignancy are all caused by a drop in HSCs, which are responsible for the synthesis of blood cells during a person's life [19]. When transplanted into a conditioned host, HSCs can repopulate the entire hematopoietic system because of their multilineage and self-renewal potential. Hematopoietic stem cells (HSCs) are derived from embryonic hemogenic endothelium (HE), a subset of ECs capable of giving birth to HSCs [20]. The BM is home to HSCs because it provides a unique milieu, often called a niche, for these cells to thrive. Hematopoietic stem cells (HSCs) and hematopoiesis are controlled by both internal and extrinsic stimuli [21]. By activating Toll-like receptors (TLRs) or other receptors that recognize the elevated levels of cytokines during infection, HSCs may react to an infection by increasing their proliferation and differentiation to satisfy the increased demand for myeloid blood cells during emergency hematopoiesis. Such cytokines include macrophage colony-stimulating factor (M-CSF), interleukin-1β (IL-1β), or tumor necrosis factor-alpha (TNF-α) that can induce the myeloid transcription factor (TF) PU.1 in HSCs to promote increased myeloid lineage differentiation [22–25]. Stem cell factor (SCF), CXC chemokine ligand (CXCL)12, and thrombopoietin (TPO) have all been shown to be essential for HSC maintenance, and there has been a great deal of study on the potential components of HSC niches. CXCL12 and SCF are produced in high amounts in BM. TPO, on the other hand, has been demonstrated to be made mainly through hepatocytes rather than bone marrow cells, suggesting that TPO primarily moves away from the liver and diffuses into marrow cavities. Based on these results, many candidate HSC niches were discovered, and important biological components of HSPC niches were revealed. These HSPC niches express certain transcription factors and cytokines crucial for the upkeep of HSCs and immune progenitor cells [26].

Human HSCs are well recognized to contribute significantly to the maintenance of antiviral immunity. Because they may develop into both myeloid and lymphoid blood cells, HSCs are called pluripotent. Monocytes, macrophages, granulocytes (neutrophils, basophils, and eosinophils), erythrocytes, megakaryocytes, platelets, and myeloid dendritic cells are examples of myeloid cells that differentiate from HSCs. T and B lymphocytes, NK cells, and lymphoid dendritic cells are components of the lymphoid cell line, produced from HSCs. HSCs are thus a crucial reserve for the differentiation and maintenance of the supply of immunocompetent cells that are functionally active [27].

## Viral infection in hemopoietic stem cell transplantation (HSCT)

Infection is a leading cause of death after hemopoietic stem cell transplantation (HSCT). Vaccination may protect against some of these diseases. Regardless of whether the recipient or donor was immunized before the transplant, most HSCT patients lose their immunity to numerous infections during the first few months following the transplant. It is safe to vaccinate with inactivated vaccines after a transplant, and doing so is an efficient strategy to restore immunity against a variety of diseases (such as the influenza virus), the risk of which is elevated due to the transplant itself [28]. HSC transplantation is a medical procedure used to treat a variety of inherited and acquired diseases. However, allogeneic HSC transplanting is restricted via an absence of appropriate donors, significant venture of illness, fatality occurring from infection caused through pathogens (bacteria, viruses, fungi, or protozoa), graft failure, and graft-versus-host disease (GVHD). However, lately, autologous HSC-targeted gene therapy has been used instead of stem cell transplanting. In this method, autologous BM or induced circumferential blood cells are prepared and refined to get a CD34 + particle. These cells are transferred by retrovirus-vectors encoding the remedial gene of essential. Subsequently, improved HSCs are injected into patients following preparative or conditioning regimens, including chemotherapy [29].

Despite advances in diagnostics, several novel prophylactic agents, the prevention and treatment of established cytomegalovirus (CMV) disease, and CMV reactivation after allogeneic hematopoietic stem cell transplantation remains a leading cause of severe and sometimes fatal infectious complications [30, 31]. Concurrent infectious problems may arise due to CMV reactivation's indirect effects, such as immunosuppression or graft failure. Despite rigorous treatment with antiviral drugs and adjuvant therapy, deadly CMV illness still accounts for as much as 45–60% of mortality in HSCT patients; CMV pneumonia and encephalitis are especially devastating. Preventative medication that actively monitors CMV from the blood of HSCT patients is a standard at the moment, with reports of improved CMV-related outcomes. Prophylaxis in high-risk populations, novel antiviral medicines, and vaccinations for CMV infection have all been the subject of clinical research during the last few decades [32]. CD14 + monocytes expressing the surface marker B7-H4 were one of the most common latency sites in the peripheral blood of healthy donors. There is some evidence that HCMV selectively infects early myeloid progenitors or supports the differentiation of infected pluripotent CD34 + cells to myeloid-lineage subsets that support latency. Reactivation leads to an increase in HCMV-specific CD4 + and CD8 + T-cells, which are likely necessary for viral suppression and/or immunity [33]. To quickly restore virus-specific immunity and prevent or cure viral infections following HSCT, adoptive transfer of donor-derived virus-specific cytotoxic T cells (VSTs) is a viable option. The infusion of donor-derived CMV or Epstein-Barr virus (EBV)-specific T cells has been shown in early proof of concept experiments to restore virus-specific immunity and manage viral infections efficiently. Recent trials have infused closely matched third-party VSTs and found response rates of 60% to 70%; this is because the absence of an immunity to the infecting virus in a naive donor is a crucial cause of failure [34]. In addition, in HSCs transplanted persons, the viral infection danger is enhanced up to immune restoration being stabilized. Therapy with standard-of-care antiviral drugs, such as Cidofovir (brand name Vistide), which is an injectable antiviral medication primarily used as a treatment for cytomegalovirus retinitis, is costly, needs to be continued for a long time, and has side effects. Instead, we can utilize Brincidofovir (CMX001), which is an empirical antiviral medicine created via Chimerix of Durham, NC, for the therapy of cytomegalovirus, adenovirus, smallpox, and ebolavirus infections. The use of this drug demonstrated positive and successful results in the treatment of a nine-year-old afterwards-HSCs transplantation girl which was infected by adenovirus [35, 36].

**Fig. 1:**
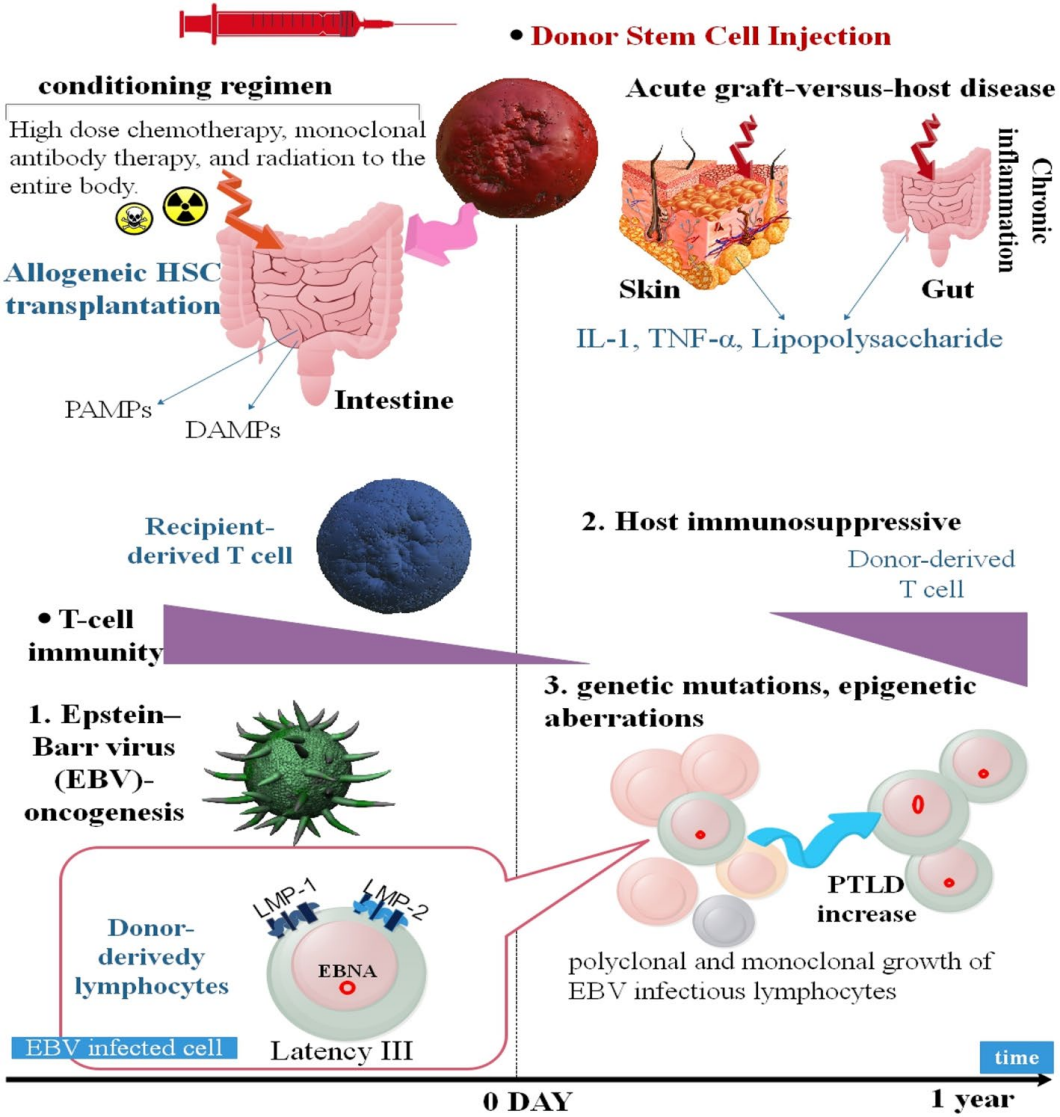
The process through which EBV-dependent post-transplant lymphoproliferative diseases emerge after the transplantation of allogeneic SCs. Latent membrane protein 1/2 (LMP 1 and LMP 2), which is altered in EBV infections and activated by primary B cells, is one of the oncogenes encoded by EBV. An important contributor to the rise in post-transplant lymphoproliferative diseases is host immunosuppression brought on by the conditioning regimen, the use of immunosuppressive agents, and development benefits from EBV-infected cells brought on by post-transplant lymphoproliferative disorders (PTLD). Continuous immune system activity, persistent inflammation, and a rise in GVHD and PTLD are all brought on by conditioning regimens. ** Damage-Associated Molecular patterns (DAMPs), Epstein—Barr virus Nuclear Antigen (EBNA)

After HSCs transplantation, the level of Epstein-Barr virus-specific cytotoxic T-cells is decreased. Disabled T-cell intercede immunity owing to pre-transplant has a significant influence on the regimen and preventative immune factors. This function lets the reproduction of Epstein-Barr virus-infect B-cells. The prolonged longevity of these cells led to the creation of different genetic mutations or epigenetic alterations, such as changes in c-MYC, B-cell lymphoma 6 (BCL6), and p53; microsatellite instability; and DNA hypermethylation. Furthermore, decreased immune system activation or efficacy, continuous immune triggers, and severe and persistent inflammation all contribute to an increase in PTLD (post-transplant lymphoproliferative disorders). Pathogen-associated molecular patterns (PAMPs), such as ingredients of the virus or other pathogens, target TLRs and trigger the innate immune response. Viral components (PAMPs) and harm-related molecular templates initiate a complex signal transduction cascade by targeting the outside and within cell domains of TLRs. This augments the TLR-intercede immune reaction and eventually causes enhance transcription of pro-inflammatory cytokines, including IL-6 and TNF-α. IL-6 and TNF-α led to chronic inflammation and drove the reproduction of polyclonal Epstein-Barr virus-infectious cells. In the HSC transplantation adjustment, the conditioning regimen include significant amount of chemotherapy and whole body irradiation, frequently led to the detriment to the intestinal mucosa by affecting pro-inflammatory cytokines including TNF-α, type I IFN, IL-1, and IL-6 (Fig. 1) [37].

Hemorrhagic cystitis (HC) caused by an adenovirus (ADV) or BK virus (BKV) is a frequent complication after allogeneic HSCT (allo-HSCT). Yoshiyuki Onda et al. conducted a phylogenetic analysis of the ADV partial sequence and assessed the prevalence and related risks of HC caused by viruses. The ages of transplant recipients ranged from 17 to 68, with 50 being the most common. Fifty-eight individuals (28%) were diagnosed with HC. ADVs were discovered in 18 cases, BKVs in 51 cases, both in 12 cases, and John Cunningham virus (JCV) was discovered in one case. There was no discernible link between any of the factors and HC. Notwithstanding, the high rates of ADV- and BKV-HC between April 2016 and September 2017 were consistent with the hypothesis of nosocomial transmission of these viruses. Sequencing the viruses' hexon, E3, and penton sections revealed seven cases of ADV type 11, two cases of ADV type 35, and three cases of a type 79-related strain. Except for one example involving a strain linked to type 79, a sequencing analysis found that these strains were almost similar within each class. Finally, based on genotyping the virus and partial sequencing of the viral genome, we described ADV-HCs that may be spread in healthcare settings. Nosocomial transmission of ADV or BKV should be considered, even though it is widely accepted that the reactivation of a dormant virus is the primary cause of viral HC after allo-HSCT [38].

## Effects of viral infections on HSCs

Rapid proliferation and differentiation of HSCs and progenitor cells, followed by mobilization to the site of infections, are only a few changes that occur in the BM compartment in response to a wide variety of microbial infections [39]. Umbilical cord blood is a valuable and rich source of HSCs. The HSCs' origins are sometimes infected with viruses, such as human adenoviruses, Epstein-Barr virus, HHV-8, HHV-7, HHV-6, varicella-zoster virus, hepatitis C virus, hepatitis B virus, and cytomegalovirus. It may even be that the blood source looks healthy for HSC transplants; however, the virus is hidden in the donor blood, and the virus infection can appear with the weakening of the immune system of the HSC recipient [40, 41]. In general, because this type of stem cell is a good host for viruses and there is a possibility that viral agents (DNA and RNA) will be hidden and not be recognizable, it can lead to viral infection in the HSC recipient. As a result, greater caution should be exercised when employing this type of stem cell therapy for viral infections. Interferons (IFNs) are a group of signaling proteins that have an important role in inhibiting viral infection by controlling HSCs. The IFNs are two types and contain type I IFN, such as IFNα and IFNβ, which targets the IFNα receptor, and the type II IFN-γ, that targets the IFN-γ receptor. Moreover, type I IFNs are persuaded via virus infection and can be produced through significant kinds of virus-infected cells. In contrast, IFN-II is induced via mitogenic or antigenic stimuli, however, it can just be generated via several immune system cells, including NK and T cells. IFN-II regulates the immune reaction against a large diversity of pathogens that occur within a cell, such as several virus infections [42, 43]. Stem cells are prepared by great inherent expression of ISGs genes (interferon-stimulated); however, remain resistant to critical IFNs signaling. As a result, this function supports stem cells against virus infection [44]. For example, using ds-RNA as a viral replication inhibitor led to an HSCs increasing rapidly in an IFNα-receptor-affiliate method [45]. In vesicular stomatitis virus (VSV) and murine cytomegalovirus (MCMV) infections, inactive LT-HSCs (long-term-HSCs) are effective and activated by IFN-I. Moreover, VSV infection activated cytokine and chemokine reactions containing IL-12(p40), IL-10, CCL2, CCL3, CCL4, etc., and caused up-expression of IL-10 receptor level on long-term-HSCs. IL-12 (p40) is generated via macrophages, neutrophils, and DCs in viral infections. Additionally, the anti-inflammatory cytokine CSIF or IL-10 (cytokine synthesis inhibitory factor) can inhibit immunopathology and increase HSC self-renewal. C–C motif chemokine ligands 2, -3, and -4 (CCL2, -3, and -4) are essential for immune cell attraction in viral infections. Remarkably, as well as in MCMV infection, IL-12 (p40), IL-10, CCL-2, -3, and -4 were recognized in the BM. Therefore, these intermediaries possibly confer long-term-HSCs operation either by direct activation or indirectly via immune cell attraction and immigration, even without functional IFNAR pathway [46].

The absence of mice models or other small animals infected with Ebola virus disease (EVD) has prevented preclinical trials of treatment for EVD infection, which is a deadly viral infection. Lüdtke et al. used hHSCs transplantion in NSG-A2 mice (nonobese diabetic (NOD)/severe combined immunodeficiency (scid)/interleukin-2 (IL-2) receptor-γ chain knockout (NSG) mice) to produce EVD mice model. Human HSCs NSG-A2 ((hu)NSG-A2) mice created whole-cell ingredients of an efficient adaptive human immune response. These mice demonstrated Ebola virus pathogenesis, such as viremia, cell and organ injury [47]. LCMV (lymphocytic choriomeningitis virus) led to a significant decrease of BM endothelial, mesenchymal stromal cell-derived factor 1 (SDF1), and therecognized as C-X-C motif chemokine-12 (CXCL12)-plentiful reticular cell. In addition, LCM virus infection the dilation of BM-sinusoidal blood vessels, which decreases blood pressure is persuaded that was followed via severe vascular repairing and significant disruption of outside a cell’s environment networks overall the BM niche. One of the most crucial harm in BM stromal is a continuous and high decrease of HPSCs and HSCs via phenotype. The surviving HSCs are also disrupt their displacement ability for a long time since LCM virus infection [48]. During LCMV-cl13 infection, IFN-α production is tightly restricted to very early and brief stages after challenge and elevated IFN-γ levels progressively phase down within four weeks. Furthermore, animals lacking CD8 T cells were completely protected from the majority of hematologic side effects and HSC loss following LCMV-cl13 infection while producing IFN-α during infection. Most notably, researchers discovered that, although not blocking entrance into the cell cycle or early losses of HSC numbers, simultaneous blockade of IFN-I and II signaling protects stroma from harm, maintains functioning, and recovers competitive fitness in the chronic stages of infection. Overall, the data suggest that: (1) IFN-α is a crucial mediator of hematological symptoms in LCMV-cl13 infections, not only through its direct impact on HSCs, but also as a critical activation signal for virus-specific CD8 T-cells, causing immunopathological destruction in HSCs and stroma; and (2) HSC niche support of the BM microenvironment is impaired, resulting in a long-term impairment in HSC activity that is essentially uncoupled from the initial proliferative burst. This might result from direct exposure to cytokines; (3) also, IFNs are not the exclusive inducers of infection-resistant HSC proliferation [49]. Through controling viral and cellular gene expression, HCMV microRNAs play crucial roles in latency and reactivation in CD34 + HPCs. RhoA, a small GTPase necessary for CD34 + HPC self-renewal, proliferation, and hematopoiesis, is the target of HCMV miR-US25-1. When miR-US25-1 is expressed, it disrupts signaling through the non-muscle myosin II light chain, preventing cytokinesis and reducing cell growth. In addition, infection with an HCMV mutant deficient in miR-US25-1 boosted CD34 + HPC proliferation and decreased the percentage of genome-containing cells at the end of latency culture [50]. Human T-cell leukemia-lymphoma virus type 2 (HTLV-II) sickness causes a wide range of immunological diseases, similar to HIV disease. This virus may have a direct influence on hemostasis in human hematopoietic progenitors. In hematopoietic precursors, HTLV-II may block apoptosis induced by IL-3 withdrawal. In addition, GM-CSF, IFN-γ, and stem cell elements were discovered to be activated by HTLV-II in an HSC line [51].

## Overview of SARS-CoV-2 characteristics

The COVID-19 pandemic has had far-reaching consequences worldwide, and the virus that caused it is still actively spreading. Emergency use authorization has been developed for monoclonal antibody-based single- and combination-therapy approaches. In addition, many other vaccine constructions have shown effectiveness, including two that provide around 95% protection against COVID-19 [52]. Approximately 30,000 nucleotides make up the SARS-CoV-2 genome, and they are split up into genes that code for structural and nonstructural proteins (Nsps). Spike (S), envelope (Env), membrane (M), and nucleocapsid (N) proteins are all examples of structural proteins. To enhance viral replication and transcription, Nsps, which are created as cleavage products of the open reading frame 1ab (ORF1ab) viral polyproteins. Assisting Nsp7 and Nsp8 in controlling viral RNA synthesis is RNA-dependent RNA polymerase, also known as Nsp12. Moreover, the ORF3a, ORF6, ORF7a, ORF8, and ORF10 genes code five auxiliary proteins [53]. Targets for antiviral intervention include the viral and cellular components involved in cell entrance since SARS-CoV-2 employs its S protein to enter target cells. Soluble ACE2 and serine protease inhibitors block host cell entrance by preventing S protein binding to the cellular receptor angiotensin-converting enzyme 2 (ACE2) and S protein priming by TMPRSS2 [54]. Both the S1 and S2 fragments are produced when S proteins are cleaved. Unlike S2, which facilitates membrane fusion, S1 has N-terminal (NTD) and receptor-binding domain (RBD) domains. Many protein-based inhibitors, including monoclonal and serum antibodies, may target the RBD due to its high level of antigenic similarity [55, 56]. SARS-CoV and Middle East Respiratory Syndrome Coronavirus (MERS-CoV) caused outbreaks of severe pneumonia, and four of them (OC43, HKU1, 229E, and NL63) are endemic and cause the common cold. Although individuals infected with any of these coronaviruses will develop antibody and T-cell responses, antibody levels will decline more quickly than T-cell responses [57]. The expression of ACE2 has been reported in various of cells, such as intestinal epithelial cells, renal tubules, endothelial cells, cerebral neurons, immune cells (e.g., alveolar monocytes/macrophages), erythroid progenitors, and HSPCs [58].

Interestingly, SARS-CoV-2 dysregulates the function of receptors involved in regulationing blood pressure, fluid and electrolyte balance, and systemic vascular resistance. At the same time, HIV uses entry receptors that are abundantly expressed on the surface of immune and hematopoietic cells (CD4, CXCR4, and CCR5). In particular, SARS-CoV-2 causes hyperactivation of the renin–angiotensin–aldosterone pathway because it exploits the ACE2 receptor for cell entry, which is internalized following viral contact [59]. According to novel research, 80% of the RBD-SD1 of SARS-CoV-2 strongly links to different BM cells, which may indicate that HSPCs are under the impact of COVID-19 in the BM niche. So, COVID-19 has an effect on the hematopoietic system [58, 60].

### SARS-CoV-2 infection in HSCs

SARS-effects of CoV-2 on HSCs and HPCs may have significant implications for immunological responses, hematologic problems, and stem cell-based treatments such as HCT. The SARS-CoV-2 infection can potentially impair hematopoiesis or directly affect immune cells, both of which may lead to dysregulated immunological responses and other hematologic problems; understanding how this virus could affect HSCs/HPCs and immune cells is thus crucial. Since many COVID-19 patients have presented with lymphopenia and thrombocytopenia, there seem to be SARS-CoV-2-caused effects on primitive and/or mature blood cell populations [61]. CD13 is an adhesion molecule expressed upon the faces of BM CD34^+^ cells, megakaryocyte cell line M-07e, and platelets, and exists on the faces of human BM CD34^+^ stem cells, platelets, megakaryocytes, and EACAMla (CD66a). CD13 is a possible receptor for the internalization of SARS-CoV-2 in CD34^+^ cells and MK cell lines, followed by quick virus replication and apoptosis. So, the COVID-19 virus can immediately target HSCs or hematopoietic progenitor cells [62]. The NLRP3 inflammasome is a multimeric protein complex that induces an inflammatory response, causes cell death, and activates the secretion of pro-inflammatory cytokines such as IL-1β and IL-18. ACE2 receptors and angiotensin II receptors are expressed and functional on the surface of HSPCs. Thus, SARS-CoV-2 can cause an infection pool of HSPCs without requiring any other factors, and pathological triggers of the Nlrp3 inflammasome can cause a cytokine storm and pyroptosis of HSCs [63, 64]. Sumorejo et al. demonstrated an increasing level of HSCs in the infected cells, which are created via planting SARS-CoV-2 in rat kidney cells and Vero cells. In each of the different infection quantities, HSCs resulted in a reduction of infected cells in 24–72 h [65]. Utilizing stem cells to express antiviral IFN-induced genes is a complicated method. β-glucan can induce HSCs and myeloid progenitors. Therefore, Chen et al. used the cold atmospheric plasma (CAP) method to treat SARS-CoV-2 infection. CAP method improves DCs maturation in the lymph node. DCs may exist as the main histocompatibility complex peptide in T cells. T cell-interceded immune reaction may be completed via the immune checkpoint suppressor and increase local and systemic antiviral immunity [66].

Patients may have catastrophic illness progression, severe thrombocytopenia, acute inflammatory anemia, and systemic thrombosis, indicative of further SARS-CoV-2 interferences on HSCs throughout the differentiation process towards erythroid and megakaryocytic cells. Therefore, through the ACE2 entry pathway in primary CD34 + HSCs, the SARS-CoV-2 RNA replicates, proteins are translated, and infectious particles are formed as the S proteins in hematopoietic cell lines. This results in the ex vivo formation of defective erythroid and megakaryocytic cells that are eventually targeted by humoral and adaptive immune cells. The greatest danger of severe thrombosis-transmitted SARS-CoV-2 infections comes from the viral particles released by infected CD34 + HSCs or the cellular component of RBC units and, ultimately, platelets [67]. As a result, it is possible that the SARS-CoV-2 direct effect on HSCs/HPCs is responsible for many of the hematologic symptoms of COVID-19 described.

**Fig. 2:**
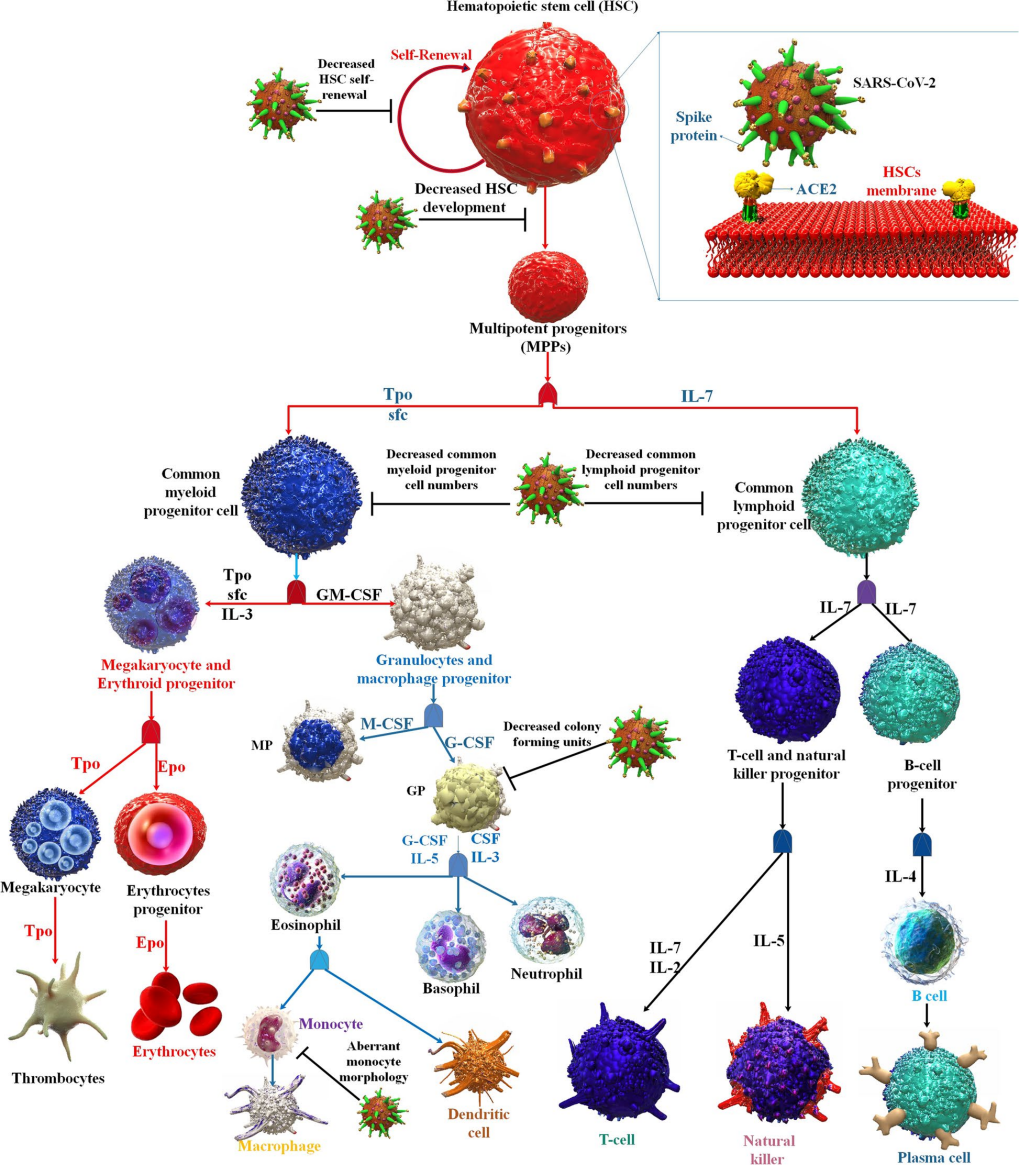
The impact of SARS-CoV-2 on the development of bone marrow-derived hematopoietic stem/progenitor cells into lineage-specific blood cells is shown schematically below. Colony-forming units (CFUs), common myeloid progenitors (CMPs), and common lymphoid progenitors (CLPs) are all multipotent progenitors derived from HSCs, which in turn create oligopotent progenitors, unipotent progenitors, and eventually fully differentiated cells in a self-renewing cycle. To increase monocytes, macrophages, granulocytes, and megakaryocytes, platelets, and erythrocytes, the CMP may produce granulocyte–macrophage progenitors (GMP) and megakaryocyte/erythrocyte progenitors (MEP). Pro-erythroblast colony forming unit-erythroid (CFU-E) arise from erythroid burst organizing unite (BFU-E), from which erythrocytes are generated; and an increase in CLP caused a transition from B-cell and T-cell progenitor to mature B-cell and T-cell lymphocytes. More so, certain HSCs contain the ACE2 receptor, and being exposed to SARS-CoV-2 S protein causes activation of inflammatory response genes. HSCs, MPP, CMP, GMP, MEP, MLP, NK cell, T cell, B cell, and monocyte all have ACE2 receptors

Thrombocytopenia and lymphopenia are two common hematologic side effects brought on by COVID-19 [68, 69]. James Ropa et al. found that ex vivo treatment of HSCs/HPCs with SARS-CoV-2 S protein dramatically reduced the potential for the generation of Multilymphoid Progenitor (MLP). Since MLPs are progenitors that differentiate into T-cells, B-cells, and NK cells, lowering the HSCs and multipotent progenitors (MPPs) capacity to develop into lymphoid-primed cells may help to explain why COVID-19 patients have fewer lymphocytes than healthy individuals. Further, researchers also noticed a decline in the quantities of colony-forming unit-granulocyte–macrophage (CFU-GM) and CFU-GEMM cells when CD34 + cells were expanded ex vivo in the presence of S protein, as well as a decline in the growth of CMP/MEP/CFU-GEMM/CFU-GM. Therefore, it's plausible that the observed decreases in cells that give rise to megakaryocytes might help partly explain why there are fewer circulating platelets in COVID-19 patients [61] (Fig. 2).

Recipients of allogeneic HSCT represent the most challenging subgroup of immunocompromised individuals. Usually, it takes 4–6 months following HSCT before the immune response is restored. Immune reconstitution may also be delayed by conditions such as GVHD and long-term immunosuppressive drugs. Therefore, in the first months after HSCT, patients have significant secondary immunodeficiency and a meager chance of developing a pathogen-specific immune response. This example illustrates researchers’ practice of administering a short course of COVID-19 to a youngster shortly after allogeneic HSCT. Immunocompromised individuals, such as HSCT patients, may do badly if they are exposed to COVID-19 due to their impaired capacity to develop an adaptive immune response to infections. Transfusion of super immune SARS-CoV-2 convalescent plasma is under investigation as a therapeutic option for severe COVID-19 [70]. SARS-CoV-2 patients who have had HSCT or CAR-T treatment are at a higher risk of experiencing adverse consequences. Compared to healthy individuals, HSCT and CAR-T recipients had a reduced immune response to SARS-CoV-2 vaccination. In addition, the humoral response in HSCT recipients was significantly influenced by the time interval between transplant and vaccination, the dose of immunosuppressive therapy (IST), and the number of lymphocytes present at immunization. Patients who received B-cell maturation antigen (BCMA)-based CAR-T had a much greater seroconversion rate than those who received CD19-based CAR-T [71].

The HSC proliferation, growth, and differentiation pathways depende on inflammatory functions as a producer of connate immune cells. In addition, HSCs express receptors necessary to detect pathogen agents, different cytokines, and their receptors. Of the cytokines generated via HSCs and PHSCs, IL-6 emerges to be significant as a controller of paracrine-style reproductions and myeloid differentiation, and as a myelopoiesis stimulator both in vitro and in vivo [72]. IL-6 can probably increase and exacerbate the symptoms of COVID-19; therefore, it is possible to utilize anti-IL-6 in the treatment of SARS-CoV-2 infection. For example, Atlizumab is an immunosuppressive drug and anti-IL6, and it is considered an off-label treatment for those with COVID-19-related acute respiratory distress syndrome. In addition, it does not decrease the levels of cytotoxic CD8^+^ and plasma B cells, so it does not prevent the powerful adaptive immune reactions. In another example, intravenous injection of Siltuximab or CNTO 328, which is a chimeric monoclonal antibody binding to IL-6, led to a decrease in the C-reactive protein measure in all SARS-CoV-2-infected cases and, importantly, improved clinical situations by decreasing the requirement for ventilation in 33 percent of COVID-19 cases [73]. Decreasing natural killer cell effector capability is the most outstanding immunological characteristic of the macrophage activation syndrome (MAS), a situation that may be activated via infections and nearly simulates the “hyperferritinemic syndrome”, which researchers contrast with COVID-19 virus-dependent on cytokine storm. Analogously to what occurs in MAS, local and systemic inflammation chips in decrease natural killer cell acts; particularly, increased IL-6 and IL-10 measures (as seen in SARS-CoV-2 infection) can repress natural killer cell cytotoxic acts as the expression of PRF1 (Perforin 1) and GrB. In addition, IL-6 can decrease NK function by reducing the expression of NKG2D, which is a transmembrane protein belonging to the CD94/NKG2 family of C-type lectin-like receptors and acts significantly in the killing of infected cells [74]. In another example, CD115 affects the differentiation and responsibility of macrophages. Also, angiotensin II receptors have appeared in the regulation of the CD115 in HSCs. Furthermore, angiotensin II regulates monocytic cells on BM stromal cells-derived TNF- to improve M-CSF-mediated control of monocytic cells. The shortage of ACE2 in BM-originated cells enhances the expression of TNF-α in adipose stromal cells. It was suggested that ACE2 expression regulates inflammation in BM cells of adipose tissue. ACE2 shortage in BM-originated cells improves atherosclerosis via the adjustment of Angiotensin II/Angiotensin-(1–7) peptides. Additionally, critical ACE prohibition demonstrated an enhancement in the N-Acetyl-Ser-Asp-Lys-Pro (AcSDKP) level in plasma, which can significantly suppress the cell cycle entrance of HSCs and support hemopoiesis versus damage caused by cycle-active cytotoxic agents. AcSDKP can also suppress the lymphocytes production, induce angiogenesis, and has antifibrotic impact in vivo. In general, ACE2 serves a defensive function by neutralizing the negative effects of Angiotensin II-induced inflammation, and we also know that the SARS-CoV-2 virus binds to ACE2 in host cells. As a result, the therapy method, which mainly leads to decreasing the Angiotensin II-induced disadvantageous influence, causes decreasing SARS-CoV-2 infection [75]. HSCs gene therapy system, including ZFNs, TALENs, and CRISPR/Cas9, which are targeted gene editing, let accurate DNA repair, has been effectively used to treat several diseases. For example, using HSCs gene therapy in treatment of HIV infection, which improves HSCs and all obtained cell kinds, will generate the anti-HIV genes without interruption [13, 76]. Therefore, utilizing HSC gene therapy may be useful and efficient in the treatment of SARS-CoV-2 infection.

## HSC gene therapy in viral infection

Disease phenotypes caused by inherited or somatic mutations in HSCs are diverse and may impact both the HSCs and the differentiated cells that arise from them. Since HSC mutations underlie many inherited hematological illnesses, their elimination would be curative [77]. Given these advancements, it is expected that it will be theoretically possible to safely modify stem cells ex vivo, transplant cells into healthy people receiving effective antiretroviral therapy without ablative conditioning (possibly using a large number of gene-modified cells or an in vivo selection approach), and carefully interrupt therapy, potentially resulting in the expansion of gene-modified cells [16, 78]. By avoiding these restrictions, autologous HSC-based gene therapy ought to be safer. A growing range of illnesses is being successfully treated thanks to advances in genetically altering HSCs via either vector gene insertion or gene editing [15]. Ex vivo HSC gene transfer is the foundation of the traditional strategy, which has shown positive outcomes. Ex vivo HSC transduction is used in two of the six gene therapy medications approved by the FDA or the European Medicines Agency (EMA): Simvelis for ADA-SCID and Zynteglo for -thalassemia, which the EMA has approved conditionally. Ex vivo HSC gene therapy isn't widely available for patients, however, especially in areas with poor resources where the most hematological illnesses are prevalent due to its high cost and adverse effects. In vivo HSC transduction, on the other hand, stands out because of its technological simplicity and low cost, and it might be administered as an outpatient therapy [79]. Researchers discovered that the CD34 + CD90 + cell fraction was primarily responsible for multilineage engraftment and hematopoietic regeneration in an animal model of primate gene therapy and transplantation. This study is the first to show that a CD34 subset enriched in HSCs may be a translational tool for lentivirus-mediated gene therapy. Separating CD133 + cells or CD38low/subsets of CD34 + cells from human blood products are two other HSC enrichment strategies. Isolating these HSC-enriched CD34 + CD90 + HSPCs may increase the targeting yield of existing clinical HSC gene therapy applications [80].

**Fig. 3:**
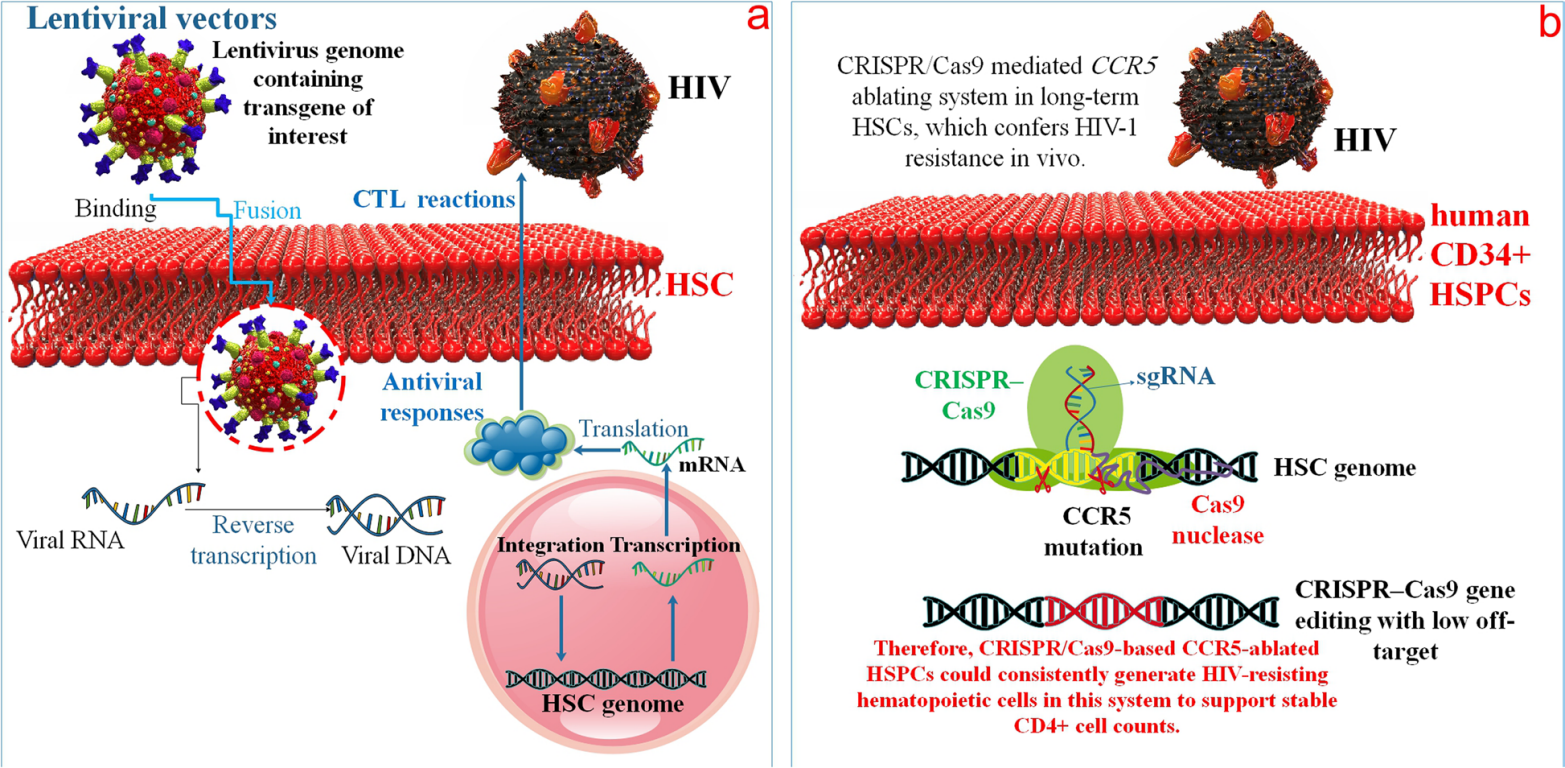
HIV-inhibiting genes that may block the virus at several stages of replication have been developed by innovative application of science; **a**) lentiviral vectors permit semi-random insertion of transgenes into the genome; and **b**) CRISPR/Cas9. The CRISPR-Cas9 system induces insertions and/or deletions in specific sections of the genome by generating a double-strand break in DNA and allowing it to be repaired through the non-homologous end-joining pathway. The Cas9 protein in the CRISPR-Cas9 system is directed to a particular target DNA sequence by a single guide RNA (sgRNA)

For example, T cell reactions, especially CTL (cytotoxic T lymphocyte) reactions, are essential in regulating and inhibiting virus replication or cancer cells development and the failure of this reaction is an important element in increasing these diseases. However, we can utilize transplanting HSCs, and genetic engineering of these stem cells causes an increasing reaction. For example, Kitchen et al. used transgenic TCRs (T cell receptors) in human HSCs, which can be utilized to bind polyclonal adult circumferential blood derivative CD8^+^ T cells to fight HIV infection. In this method, the TCR gene is introduced into a HSCs by lentiviral vector, which causes the development of a vast crowd of multifunctional, HIV-particular CD8 + cells with a ability of identifying and destroying HIV antigen cells [81] (Fig. 3). Other gene therapy methods of HIV treatment by utilizing HSC include HIV inhibitor genes able to prevent several stages of viral infection that have been produced with new applied science, such as RNA interference (RNAi), Zinc-finger nucleases (ZFNs), transcription activator-like effector nucleases (TALEN), and CRISPR/Cas9 [82]. Transplantation of HSCs with a usually occurring C–C chemokine receptor type 5 mutation, also known as the CCR5 mutation, confers a noticeable loss of HIV-1, and production ablation of the CCR5 gene, which is a method of modifying DNA to disrupt the production of a specific gene, is a perfect treatment method for HIV-1 infections. This approach was used in human CD34 hematopoietic progenitor-stem cells (HSPCs) to achieve successful CCR5 ablation for the treatment of HIV infection [83] (Fig. 3). Another gene therapy strategy involves using antiviral genes inside HSCs, such as RevM10, to offer long-term support to precursor T-cells produced from transduced stem cells to prevent HIV infection. RevM10 is a dominant-negative mutant of the HIV-1 Rev gene, which encodes an RNA-targeting protein including in the nuclear trafficking of unintentional virus mRNAs. RevM10 is able to prevent HIV proliferation in T cells isolated from transduced hematopoietic progenitor-stem cells afterward development in vitro [84]. To combat HIV using a genetic vaccination technique, engineering T cells with anti-HIV CAR has emerged as a potential strategy. Studies have shown that a shortened version of the CD4-based CAR (D1D2CAR) improves the differentiation of CAR-T cells from gene-modified HSCs while retaining the same level of CTL activity. In addition, there is less of a chance of unintended consequences with D1D2CAR since it does not induce HIV infection or stimulation mediated by IL-16. CAR-modified HSCs successfully differentiated into hematopoietic progenitors and showed enhanced antiviral action when stimulated by 4-1BB but not CD28. Some studies have demonstrated that, when added to CD4-based CARs, 4-1BB accelerated viremia suppression in untreated HIV-1 infection. D1D2CAR 4-1BB mice exhibited enhanced CAR T cell persistence and enhanced speed of viral suppression when combined with ART. Overall, the D1D2CAR-41BB outperforms the original CD4CAR in terms of HSC differentiation, viral suppression and persistence, and detrimental activities, suggesting that it should be further investigated in clinical trials [85]. While most HPCs are resistant to HIV-1 infection, when these cells develop into committed HPCs they become progressively vulnerable to HIV-1 infection, allowing viral gene expression and infectious virus generation. Due to this cellular trafficking pathway, HIV-1 replication in the BM and infection of BM HPCs may be implicated in the initial steps leading to the development of HIV-1-associated dementia (HAD). It has been demonstrated that the trafficking of BM-derived HIV-1-infected monocytes is involved in the dissemination of HIV-1 into the central nervous system (CNS). It has also been observed that HPCs in the BM of HIV-1 patients are affected by the presence of HIV-1 proteins and changes in the cytokine milieu, which may modify the maturation process and increase cell mortality within one or more BM cell lineages. As a result of HPC proliferation and differentiation, monocyte populations that are more vulnerable and/or tolerant to HIV-1 may be produced, and they may also have altered trafficking patterns to other organs, including the central nervous system. It has been shown that a monocyte subpopulation with similar characteristics, defined by low CD14 expression and the presence of cell surface CD16, expands throughout HIV-1 illness, notably in HAD patients [86].

A single administration of a gene treatment for HSCs that uses a secreted SARS-CoV-2 decoy receptor protein (sACE2-Ig) would provide lifelong protection against airway infection, viremia, and extrapulmonary symptoms. By intravenously mobilizing HSCs from the BM into the peripheral bloodstream and injecting an integrated, helper-dependent adenovirus (HDAd5/35 + +) vector system, Hongjie Wang et al. have created a novel, technically simple, and transportable in vivo hematopoietic HSC transduction technique. Researchers used vital β-globin transcriptional regulatory elements to guide sACE2-Ig expression to erythroid cells in consideration of the number of erythrocytes. Researchers used an HDAd-sACE2-Ig vector to achieve in vivo HSC transduction on CD46-transgenic mice. Treatment-treated mice had much less weight loss, decreased viremia, and reduced pulmonary pathology and cytokine production. Researchers discovered that HSCs that had been transduced in vivo gravitated toward the spleen and persisted there. Erythropoiesis and erythrocyte function was unaffected by sACE2-Ig produced from erythroid cells [18].

## Viewpoints and landscape

The hematopoiesis is accountable for high level of immune cell production that protect against viral infection and is the source of HSCs and HPCs, which are used in HCT to treat hematologic diseases. The viruses can lead to direct and indirect damage to HSPCs and the surrounding tissue [61]. Immediate pathogenic efficacy is dependent on viral tropism and replication, and there have been a few reports of direct infection of HSPCs with altered BM output. For example, SARS-CoV-2 may, on the one hand, directly infect the pool of HSPCs, and other pathological triggers of the Nlrp3 inflammasome may result in cytokine storm and pyroptosis of these cells [59]. Mature immune cells and HSCs similar can be triggered either via direct activation of pathogen recognition receptors (PRRs), including the Toll-like receptors (TLRs), or through pro-inflammatory cytokine signals. PRR induction via viruses can result in the generation of pro-inflammatory cytokines and chemokines, which can affect the growth and development of HSCs. Different cytokines, including IL-1β, IL-6, TNFα, TGFβ, M-CSF, and GM-CSF, have been recognized to control the growth and development of HSCs. Therefore, recognizing how inflammation controls HSC fate and function in typical and infectious situations shows a critical frontier for our comprehension and therapy of chronic inflammation and blood diseases. However, our understanding of the full breadth by which viral infections influence hematopoiesis remains restricted. Therefore, it is crucial to better identify the relevant cellular and molecular players in this procedure and their intricate interplay to effectively treat or prevent blood diseases and BM disorders in patients with viral infections [7, 87].

Viral infections remain the main reason for morbidity and fatality afterward allogeneic HSCT. Several viral infections in HSCT recipients arise from different types of viruses, such as VSV, MCMV, HIV, BKV, EVD, LCM, and SARS-CoV-2. The detection of the viral etiology needs clinical diagnosis of infection and laboratory assessment, which can use novel molecular methods such as non-coding RNAs and microRNAs [56, 88–91]. Hence, detection is the early method toward the overarching aim of viral infection control and inhibition. In addition, the application of preventive pharmacotherapy is efficient in decreasing the danger of some viral infections. Still, curative elections for breakthrough infections are complex by toxicities, and for many viral infections, there are restricted/no efficient prophylactic or curative pharmacotherapies [34]. The presentation of a novel type of nanoparticles with numerous special attributes and roles has led to a series of innovative uses. Nanoparticles are part of therapeutic, diagnostic, and preventive methods to control viral infections. Consequently, prophylactic agents, early detection, and treatment of viral infection by nanoparticles in HSCT recipients are critical [92–95]. In addition, gene therapy for viral infections using HSC can prevent several stages of viral infections. This treatment method has been used in HIV and SARS-CoV-2; however, no study has been conducted on other viral infections, which can help in the treatment and success of HSCT.

## Conclusion

Clinical symptoms of chronic viral infections typically occur at the level of blood cell development. Viral infections can potentially harm HSPCs and the tissue around them directly and indirectly. There have been a few instances of direct infection of HSPCs that resulted in altered BM production, and direct pathogenic effects are dependent on viral tropism and the viral cycle. Understanding these mechanisms might aid in understanding BM repair in various circumstances, such as those after radiation or chemotherapy. Because various viruses have been shown to specifically target primitive HSCs, they have the potential to act as a vector for delivering foreign genes to this cell type, underscoring the need for more research to properly describe the relative impact of multiple viral infection effects on HSCs.

SARS-CoV-2 receptors have been found on the surfaces of HSPCs, endothelial progenitor cells, and even more on immature hematopoietic and endothelial progenitors. There is also concern that the virus may target stem cells in the lungs, heart, and intestines. SARS-CoV-2 infection and mortality are both increased in persons with hematological illnesses such as leukemia, lymphoma, and autologous or allogeneic HSCT, according to recent research. The optimum approach to solving this issue has to be defined, which can only be done through further research (Table 1).

**Table 1:** Impact of viral infection in HSCs

**Viral infection**	**viral infection effect on HSCs**	**Ref**
**VSV**	Long-term HSCs show increased expression of the IL-10 receptor after infection with VSV. The anti-inflammatory cytokine human cytokine synthesis inhibitory factor (CSIF; also known as IL-10) may reduce immunopathology while simultaneously boosting HSC self-renewal	[46]
**MCMV**	It was shown that the BM expressed MCMV-specific IL-12(p40), IL-10, CCL-2, -3, and -4. Consequently, even in the absence of an effective IFNAR route, these mediators may confer long-term-HSCs function by direct activation or indirectly through immune cell attraction and immigration	[46]
**HIV**	When the TCR gene is delivered into HSCs via a Lentiviral vector, the HSCs differentiate into a large number of CD8 + T cells with the ability to recognize and kill HIV antigen cells	[81]
	CRISPR/ Cas9 gene editing methods are used in human CD34 HSPCs and attained effective CCR5 ablation for the treatment of HIV infection	[83]
	T cells derived from transduced HPSC may be inhibited in their ability to proliferate HIV by using RevM10	EVE
**EVD**	The NSG-A2 mice, which were genetically modified to include human HSCs, generated the essential cell components for a fully functional adaptive human immune response. These mice exhibited pathogenesis typical of Ebola virus infection, including viremia, cell and organ destruction, and a high degree of time sensitivity	[47]
**LCM**	The loss of HPSCs and HSCs, as measured by phenotypic, is one of the most significant negative changes in the BM stroma. Additionally, LCMV infection has a long-lasting effect on the surviving HSCs, impairing their capacity to displace other cells	[48]
**SARS-CoV-2**	Evidence suggested that Ang II was responsible for controlling CD115 in HSCs. Reducing the deleterious impact induced by Ang II has on SARS-CoV-2 infection	[75]
	Pathological stimuli of the Nlrp3 inflammasome could produce cytokine storm and pyroptosis in HSCs, and the SARS-CoV-2 may infect a pool of HSPCs independently and directly	[63, 64]
	Infected cells generated by planting SARS-CoV-2 in rat kidney cells and Vero cells showed a decline in infection after 24 to 72 h, regardless of the initial infection dose	[65]
	HSCs and myeloid progenitors may be stimulated by β-glucan	[66]

## Data Availability

Not applicable.
